# AOSR joins forces with ***biij***

**DOI:** 10.2349/biij.2.4.e44

**Published:** 2006-10-01

**Authors:** KR Thomson

**Affiliations:** Department of Diagnostic Radiology, University of Melbourne, Australia

The Asian Oceanian Society of Radiology (AOSR) has joined forces with the Biomedical Imaging and Interventional Journal (***biij***).

For many years, the AOSR has produced its own journal in text form. This journal was entirely due to the enormous energy and hard work freely given by Dr. Sudarshan Aggarwal. He was responsible for much of the financing of the Journal and due to his influence the Journal was listed by Excerpta Medica.

However, as time went by it became obvious to the Executive Council of the AOSR that the cost in terms of time, money and energy in producing the AOSR Journal could not be continued without a significant financial drain on the society.

For some time we have been looking for alternatives and one option which we considered was to conjoin the AOSR Journal to another more widely published Journal. Unfortunately none of the alternatives were entirely satisfactory because of the particular needs of our region and the enormous cost of postage.

The Executive Council has the view that in this electronic age the AOSR should establish a firm presence on the World Wide Web and thanks to Prof. Basri J.J. Abdullah it has proved possible to transfer our AOSR Journal to electronic media.

Those of you who are reading this editorial will have already seen what a great opportunity the ***biij*** format provides for the AOSR.

A most successful Congress (the 11th Asian Oceanian Congress of Radiology) was recently held in Hong Kong and brilliantly organised by Dr. Lillian Leong, the chairperson of the organising committee and the outgoing past president of the AOSR.

The theme of the meeting was “Radiology: from nano to cosmos” and the quality of the invited presentations and proffered papers was as good as, or better than, any international meeting I have ever attended.

One of the highlights was the Satyapal Aggarwal Memorial lecture which was given by Prof. Hedvig Hricak. Her lecture on advances in computed tomography (CT) was inspirational. The abstracts of the presentations will be available through ***biij***.

We should be very proud of our young radiologists who presented their research and demonstrated to the world that the Asia-Pacific region is well poised to take advantage of the rapidly changing medical paradigm.

I was very honoured to be elected as the President of the AOSR until 2008 when the 12th Congress will be held in Seoul, Korea. Over the next two years I shall represent the society at as many international meetings as possible and endeavour to visit as many of our 23 national societies as possible.

The world continues to change rapidly and we must advance to keep pace with it. Economically the Asia Pacific region is a powerhouse and we must ensure that our medical care and radiology in particular keeps pace with developments in our region. We have many more challenges to face than the other great international societies but I sincerely believe that we are more than equal to the task before us.

One of the biggest challenges is the enormous geographical scope of our region and the vast differences in the provision of medical care across the Asia-Pacific. The aim of AOSR is to ensure that radiology expands to meet these needs and joining forces with ***biij*** is the first step of a long journey.

